# Clinical importance of S100A9 in osteosarcoma development and as a diagnostic marker and therapeutic target

**DOI:** 10.1080/21655979.2019.1607709

**Published:** 2019-05-06

**Authors:** Yongliang Liu, Gongzeng Luo, Dongyong He

**Affiliations:** a Department of Trauma Orthopedics, Linyi Central Hospital, Yishui, Shandong, China; b Department of Internal Medcine, The Affiliated Hospital of Qingdao University, Qingdao, Shangdong, China

**Keywords:** Diagnostic marker, Gene therapy, osteosarcoma, short interfering double-stranded RNAs, S100A9

## Abstract

**Objective**: S100A9 is a calcium- and zinc-binding molecule of S100 family. The aim of the study was to evaluate the role of S100A9 in osteosarcoma (OS) and its value as a diagnostic and therapeutic target in OS. **Methods**: Quantitative real-time polymerase chain reaction (qRT-PCR), immunohistochemistry and microdissection-based mRNA analysis were used to detect S100A9 mRNA and protein expression in OS and normal bone tissues and its potential as a diagnostic marker in OS. *In vitro* experiments with RNA interference were performed to evaluate the functional role of S100A9 and its potential as a therapeutic target in OS. **Results**: S100A9 mRNA levels were significantly higher in OS tissues than that of in normal bone tissues. Receiver operating characteristic curves showed that S100A9 could be a useful diagnostic marker in OS. *In vitro* data showed that inhibition of S100A9 decreased the proliferation and invasiveness of OS cells, and these findings were supported by microarray data. **Conclusions**: Assessment of S100A9 mRNA expression is a promising tool for the diagnosis of OS, and S100A9 may be a promising therapeutic target in OS.

## Graphical Abstract

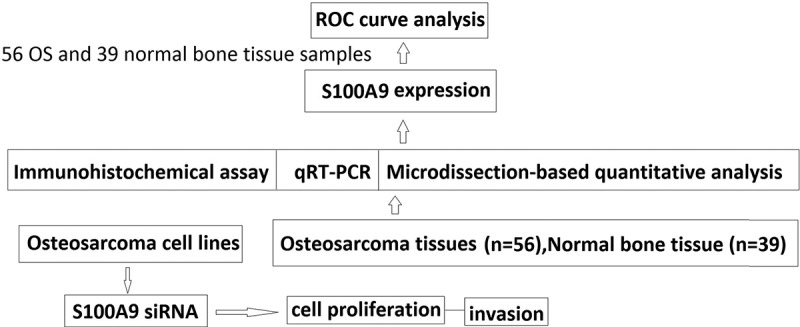

## Introduction

Osteosarcoma (OS) is the most common malignant bone tumor in children and adolescents. The 5-year survival of patients with localized OS remains at 60–70% after treatment with surgery and multi-agent chemotherapy [1]. Up to 20–25% of the patients have detectable metastatic disease at the time of diagnosis [2]. Metastatic OS is associated with poor response to standard chemotherapy, resulting in poor prognosis. The lack of useful biomarkers is an important clinical problem in OS [3]. Despite recent advances in the treatment of OS, the clinical outcomes of OS patients have not substantially improved in over 30 years [4]. Therefore, it is imperative to explore new prognostic indicators and novel therapeutic approaches for this disease once it has been detected.

S100A9 is a small calcium (Ca^2+^)-binding protein belonging to the S100 family [5]. Increasing evidence supports the important role of S100A9 in tumorigenesis. S100A9 is upregulated in colon, gastric, bladder, pancreatic, ovarian, breast, thyroid, and skin cancers, and is associated with an aggressive and invasive phenotype [6]. S100A9 promotes metastasis in melanoma [7], and knockdown of S100A9 suppresses proliferation in glioma stem cells and inhibits the growth of xenograft tumors *in vivo*, suggesting an association between the tumorigenic potential of cancer stem cells and S100A9 upregulation [8]. The expression of S100A9 is increased in human OS tissues and positively correlated with clinical classification and survival rate [9]. Taken together, these data suggest that S100A9 is a promising diagnostic marker and/or therapeutic target in OS.

In the present study, we quantitatively measured the expression of S100A9 mRNA in OS cell lines and tissues using real-time PCR. S100A9 mRNA levels were significantly higher in tumor specimens from patients with OS than in non-neoplastic tissues. Immunohistochemical studies and microdissection-based quantitative analysis of mRNA in normal bone tissue and OS were performed to investigate the role of S100A9 in carcinogenesis and the usefulness of S100A9 mRNA quantification for the detection of OS. In addition, RNA interference (RNAi) was used to evaluate the functional role of S100A9 and its possible therapeutic implications, and the effect of S100A9 knockdown on the proliferation and invasiveness of OS cells was evaluated *in vitro*. Finally, screening of oligonucleotide microarrays to identify S100A9-related transcripts detected several cancer-related genes whose expression was upregulated or downregulated by S100A9 knockdown.

## Materials and methods

### Cell lines

The human OS cell lines MG63, SaOS2, U2OS, HOS, MNNG/HOS, and 143B were purchased from the American Type Culture Collection (Shanghai, China). SaOS2 were routinely cultured in DMEM/F-12 medium supplemented with 10% FCS. MG63 and U2OS cells were grown in α-MEM (GIBCO, Grand Island, NY, USA); HOS,143B and MNNG/HOS cell lines were cultured in RPMI-1640 (Life Technologies, Grand Island, NY, USA). They were all supplemented with 10% FBS and antibiotics (penicillin 100 U/ml, streptomycin 100 lg/ml) in 37°C humidified atmosphere with 5% CO_2_.

### Patients and specimens

This study was approved by the ethics committee of Yantai Yuhuangding Hospital, China, and all patients provided informed consent. The study was conducted with the approval of the Yuhuangding Hospital Institutional Review Board (YHD 20130719). A total of 56 OS tissues and 39 normal bone tissue samples were obtained at the time of surgery between 2009 and 2017 at Yuhuangding Hospital, Yantai, China. The tissue samples were removed as soon as possible after resection, embedded in ornithine carbamyl transferase compound, snap-frozen for analysis by microdissection, and stored at −80°C or fixed in formalin and embedded in paraffin for immunohistochemistry. All tissues adjacent to the specimens were examined histologically, and the diagnoses were confirmed. Metastasis was defined as the presence of metastatic disease at the time of initial diagnosis. Recurrence was defined as the presence of recurrence at the last follow-up visit of the patient.

### Quantitative assessment of S100A9 mRNA levels by real-time PCR

Total RNA was extracted from cultured cells and isolated cells using a microdissection technique with a PicoPure RNA Isolation Kit (Arcturus, Mountain View, CA, USA) or High Pure RNA Isolation Kit (Roche Diagnostics, Mannheim, Germany) with DNase I (Roche Diagnostics) treatment according to the manufacturer’s instructions. The primers used were as follows: S100A9, 5ʹ-AGTCGAGCTAG CAAACACTCTGTGTGGCTCCTCG-3ʹ and 5ʹ- CTAGTACTCGAGC GTCTTGCACTCTGTCTGTGTAAT-3ʹ; GAPDH, 5ʹ-CTTTGGTATCGTGGAAGGACTC-3ʹ (forward) and reverse 5ʹ-GTAGAGGCAGGGATGATGTTCT-3ʹ. qPCRs were performed using the SYBR® Select master Mix (Thermo Fischer Scientific, Erembodegem, Belgium) in a QuantStudio 12K Flex real-time PCR machine (Thermo Fischer Scientific). The cycling protocol was as follows: 3  min at 50°C and 3  min at 95°C, followed by 40 cycles of 3  s at 95°C and 30  s at 60°C. Gene expression was presented as the ratio of the expression of each target gene mRNA to that of β-actin mRNA.

### Immunohistochemical assay

Serial 4 μm-thick sections were pre-treated by microwave heating in 10 mM citric acid buffer for 4 × 5 min. The sections were immersed in 0.6% H_2_O_2_ in methanol for 20 min at room temperature to block endogenous peroxidase activity. After blocking non-specific protein binding by overnight incubation with Block Ace, the sections were incubated with primary antibodies against human S100A9 overnight at 4°C. Subsequently, sections were incubated with secondary antiserum (1:500) for 1 h, followed by incubation with peroxidase anti-peroxidase complex for 30 min at room temperature. The sections were then washed with water and counterstained with hematoxylin. Immunocomplexes were visualized using stable 3,3V-diaminobenzidine tetrahydrochloride. The sections were rinsed with distilled water and counterstained with hematoxylin for 10 s. Immunohistological results were assessed by two authors. The staining result for S100A9 was subdivided in negative expression (cell staining < 10%) and positive expression (cell staining ≥ 10%).

### Microdissection-based quantitative analysis of S100A9 mRNA

Frozen tissues were cut into 8-μm-thick sections. One section was stained with H&E for histologic examination. Cells from 56 OS tissues and 39 normal bone tissue samples were selectively isolated using laser microdissection and a pressure catapulting system in accordance with the manufacturer’s protocols. After microdissection, total RNA was extracted from the selected cells and subjected to real-time RT-PCR for quantitative measurement of *S100A9.*

### Inhibition of S100A9 mRNA by small interfering (si) RNA

Two S100A9-targeting siRNAs (siRNA1: target sequence AAGCCCTCAAGGGCTGAAAAT; siRNA2: target sequence AAGCTGCAGGATGCTGAAATT) were purchased from Cell Signaling Technology, Inc (Shanghai China). To verify the specificity of the knockdown effect, U2OS and MG63 cells were transfected with the indicated 100 pmol siRNA using Nucleofector according to the manufacturer’s instructions. Briefly, 3 × 10^4^ cells/ml were plated in 6-well plates. Twenty-four hours later, 100 pmol of siRNA was mixed with HiPerFect transfection reagent (Qiagen) at 1:3 ratio (siRNA: transfection reagent). The mix was incubated 10 min at room temperature (RT) and then added to the cells. Cells were collected 48-hr later to assess the downregulation of each target.

### Cell viability assay

Cells were transfected with 100 pmol of the indicated siRNA and seeded in 24-well plates at a density of 5 × 10^4^ cells per well. The MTT (3-(4, 5-dimethylthiazol-2-yl)-2, 5-diphenyltetrazolium bromide) assay (Sigma-Aldrich) was performed to measure the effect of S100A9 siRNA and control siRNA on cell proliferation using previously described methods [10].

### Cell invasion assay

Cell invasion experiments were performed using the Bio-Coat cell migration chamber (BD Biosciences, Massachusetts, Boston, USA), which consists of a 24-well companion plate with cell culture inserts containing an 8 μm-diameter pore filter. Cancer cells were transfected with the indicated siRNA and seeded in the Matrigel-coated Transwell inserts. After 48 or 72 h of incubation, cells invading the lower surface of the Matrigel-coated membrane were counted under a light microscope (QImaging Retiga 4000R).

### Oligonucleotide microarray expression assay

The human 1A microarray (Agilent Technologies) contains *in situ* synthesized 60-mer oligonucleotides representing 17,086 unique human genes. The probes on these arrays were designed primarily for 3ʹ regions of expressed sequences in the human genome. Signal intensity for each transcript (background subtracted and adjusted for noise) and detection call (present, absent, or marginal) were determined using Microarray Suite Software 5.0 (Affymetrix).

### Statistical analysis

Data of clinical samples were analyzed by the Mann–Whitney U test. The ability of S100A9 expression to differentiate carcinoma from non-neoplastic disease was assessed using ROC curves constructed by calculating the sensitivities and specificities of S100A9 data at several predetermined cutoff points. Statistical analysis of the differences in the receiver operating characteristic (ROC) was performed using SOSS 22.0. Differences in immunohistochemistry data were analyzed with the Kruskal–Wallis test if comparisons involved three groups and the Mann–Whitney U test if comparisons involved two groups. For *in vitro* experiments, values were expressed as the mean ± SD. Student’s t-test was used for differences between two groups. P < 0.05 was considered statistically significant for all tests.

## Results

### Quantitative analysis of S100A9 mRNA levels in cancerous and non-cancerous bone tissues

**Figure 1.**
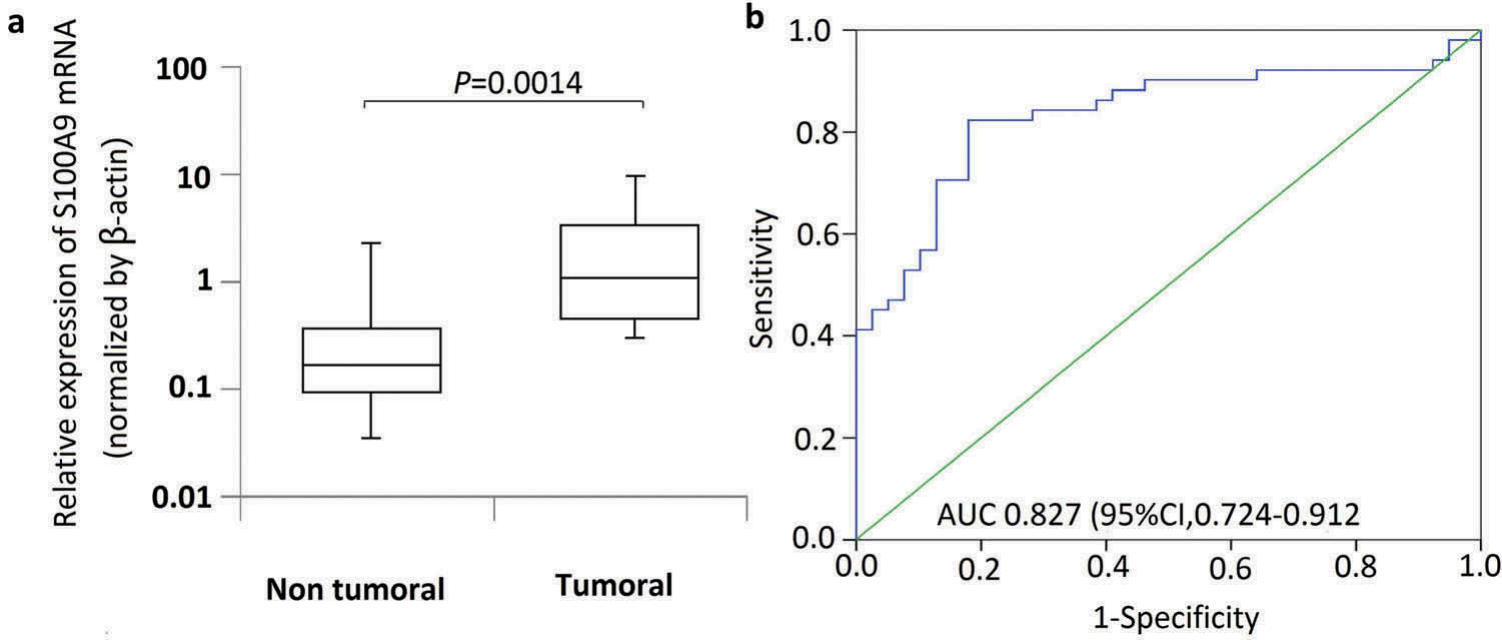
S100A9 mRNA levels in 56 osteosarcoma tissues and 39 normal bone tissue samples. The relative expression level of S100A9 was normalized to b-actin. (a) Box plot of S100A9 expression levels in osteosarcoma tissues and normal bone tissues. The boxes indicate the 25th and 75th percentiles, and the bold lines represent the median values. The relative expression levels of S100A9 were normalized to b-actin. Statistically significant differences were determined using Mann–Whitney tests or Kruskal–Wallis tests. B. Receiver operating characteristics (ROC) curve analysis of the diagnostic value of S100A9.

S100A9 mRNA levels were measured in 56 OS and 39 normal bone tissue samples. β-actin was used as a reference gene to quantify target gene expression (Figure 1(a)). S100A9 expression was significantly higher in tumor than in normal bone tissue samples (P = 0.0014). ROC curve analysis was performed to assess the ability of S100A9 to discriminate between tumor and normal bone tissues. The area under the ROC curves of S100A9 for tumor and normal bone tissues was 0.827 [95% confidence interval (CI), 0.724–0.912] (Figure 1(b); P = 0.008).

### Differential expression of S100A9 in OS and normal bone tissues

**Figure 2.**
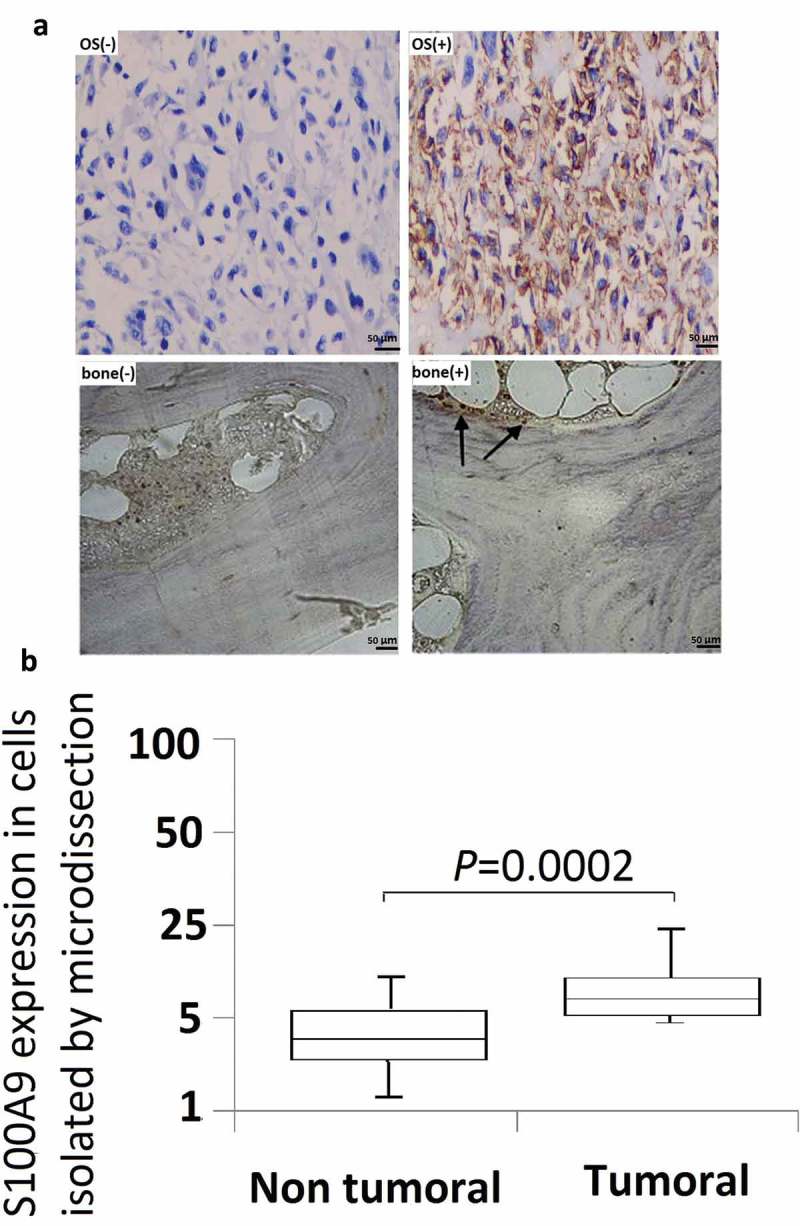
Immunohistochemistry studies and microdissection-based quantitative analysis of S100A9. (a) Immunoreactivities of S100A9; representative images of positive and negative S100A9 expression in normal bone tissues; representative images of positive and negative S100A9 expression in OS tissues. Original magnification, × 200. (b) A microdissection technique was used to isolate normal bone tissues and OS tissues from frozen sections, and total RNA extracted from these cells was subjected to quantitative analysis of S100A9 mRNA using real-time PCR. Differential expression of S100A9 mRNA was observed between normal bone tissues and OS tissues (P = 0.0002).

To confirm that quantification of S100A9 mRNA is a valid method for differentiating OS from normal bone tissue, the expression of S100A9 mRNA and protein was compared between OS and normal bone tissues. Representative immunohistochemistry data obtained with the anti-S100A9 antibody are shown in Figure 2(a). S100A9 was positively expressed in 67.8% (38/56) of OS samples, whereas positive S100A9 immunoreactivity was observed in 20% (8/39) of normal bone tissues.

A microdissection technique was used to isolate normal bone cells or OS cells from frozen sections, and S100A9 mRNA expression was quantified in these cells. S100A9 mRNA expression was higher in OS cells than in normal bone tissues (Figure 2(b)), and the difference was statistically significant (P = 0.0002).

### S100A9 mRNA expression in six OS cell lines

**Figure 3.**
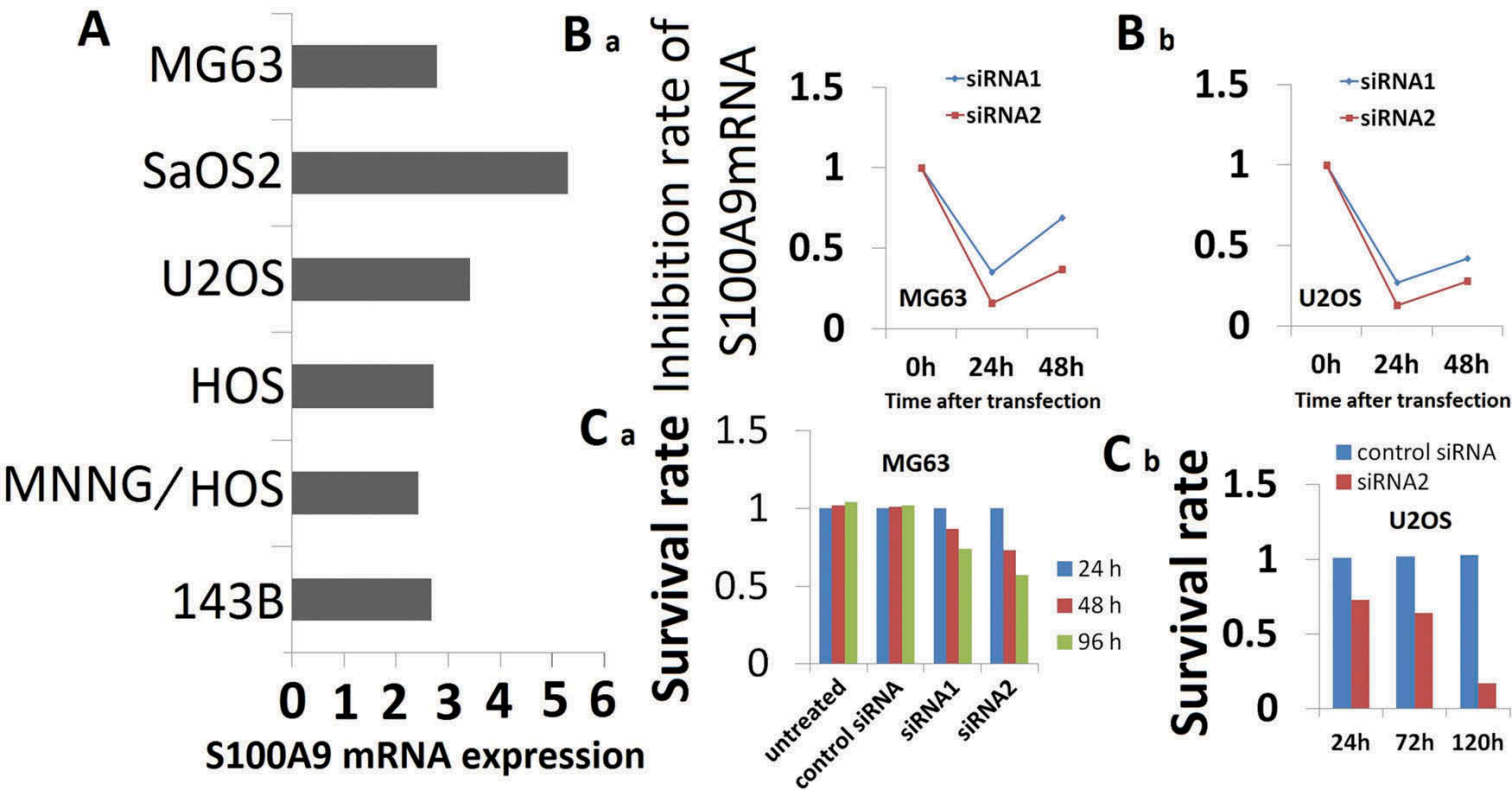
Effect of S100A9 silencing on cell proliferation. (a) S100A9 mRNA expression in 6 OS cell lines. Expression of S100A9 mRNA was normalized to that of b-actin mRNA. All six OS cell lines were positive for the expression of S100A9 mRNA. The median value was 2.6. MG63 and U2OS cells showed moderate expression of S100A9 mRNA. (b) RNAi induced inhibition of S100A9 mRNA. Two siRNAs against S100A9 were designed (siRNA1 and siRNA2). siRNA (100 pmol) was transfected into cancer cells with the Nucleofector reagent. S100A9 mRNA expression was evaluated in MG63 (a) and U2OS (b) cells at 24 h (a) and/or 48 h (a and b) after transfection. Both siRNAs showed efficient inhibition of transcript expression. Inhibition by siRNA2 was stronger than that by siRNA1. (c) Effect of inhibition of S100A9 mRNA on the proliferation of MG63 (a) and U2OS (b) cells. Cells were seeded after transfection of siRNA and evaluated by MTT assay on days 1, 2, and 4 (a) or on days 1, 3, and 5 (b) after transfection. The proliferation of MG63 and U2OS cells was inhibited. The degree of inhibition of cell proliferation was correlated with that of S100A9 mRNA in MG63 cells.

S100A9 mRNA expression was measured in six OS cell lines, namely, MG63, SaOS2, U2OS, HOS, MNNG/HOS, and 143B, and cell lines appropriate for *in vitro* RNAi experiments were selected. As shown in Figure 3(A), all six OS cell lines expressed S100A9 mRNA. The median expression was 2.6. For subsequent experiments, we selected MG63 and U2OS cells because they expressed moderate levels of S100A9 and because the conditions for siRNA transfection with nucleofector were previously established in these cells.

### Effect of inhibition of S100A9 on cell proliferation and invasion

RNAi assays were used to investigate the potential of S100A9 as a therapeutic target in OS. The degree of inhibition of S100A9 mRNA expression induced by siRNA against S100A9 is shown in Figure 3(B). S100A9-targeting siRNA1 and siRNA2 inhibited S100A9 mRNA levels in MG63 cells to 35% and 70% of the control, respectively, at 24 and 48 h after transfection (Figure 3(Ba)). Similar results were obtained in U2OS cells at 48 h after transfection (Figure 3(Bb)).

**Figure 4.**
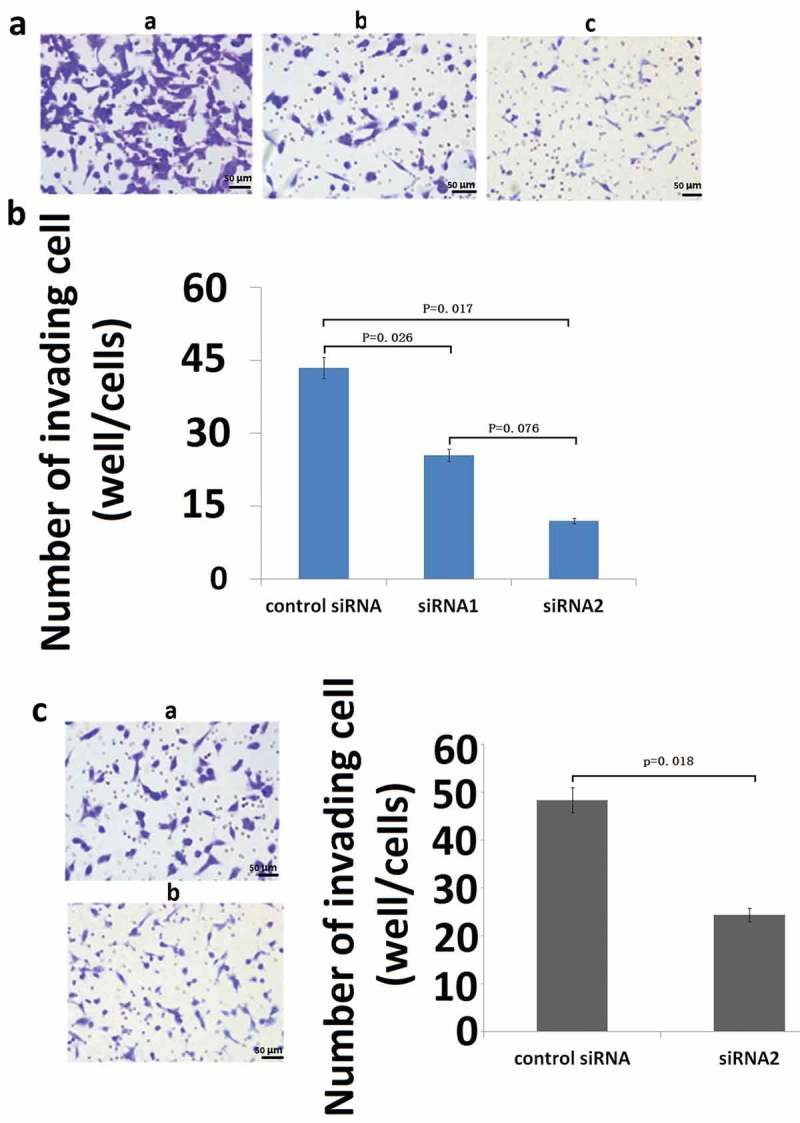
Effect of inhibition of S100A9 mRNA on the invasive potential of MG63 (A and B) and U2OS (C) cells. For invasion assays, cells transfected with siRNA were seeded in a cell migration chamber with the inner well coated with Matrigel (40 ug for MG63, 20ug for U2OS) and incubated for 48 h (MG63) or 72 h (U2OS). The number of cells invading through the Matrigel-coated membrane was then counted. (a) Representative photomicrographs of MG63 cells treated with control siRNA (a), siRNA1 against S100A9 (b), and siRNA2 against S100A9 (c). Original magnification, ×100. (b) There were significant differences in the number of invading cells between the groups (P = 0.026, control siRNA versus siRNA1 against S100A9; P = 0.017, control siRNA versus siRNA2). (c) Representative photomicrographs of U2OS cells transfected with control siRNA (a) or siRNA2 against S100A9 (b). Original magnification, ×100.There was a significant difference between the two groups (P = 0.002).

Inhibition of S100A9 expression in MG63 cells significantly suppressed cell proliferation in an inhibition rate-dependent manner 4 days after transfection (Figure 3(Ca)). In U2OS cells, inhibition of S100A9 expression suppressed cell proliferation at 5 days after transfection (Figure 3(Cb)).

**Table 1.** List of nine upregulated genes after transfection of siRNA against S100A9.

Fold change	*p*	Genename (symbol)	Putative cellular function
7.236	0.002	TNFa-induced protein 3 (TNFAIP3)	Inhibit NF-kB activation and TNF-mediated apoptosis
5.362	0.016	Inhibin beta A (INHBA)	Invasion and tumorigenesis
5.043	0.0027	Protein phosphatase 2A (PP2A)	Cell cycle
4.85	0.018	pro-inflammatory IL-6	Anti-apoptotic effect
4.25	0.046	Heparan sulfate D-glucosaminyl 3-Osulfotransferase1precursor	The heparan sulfate biosynthetic enzyme family
3.17	0.007	cytokine gro-β	Antiproliferative effect
2.98	0.025	Selectin E precursor cell	Adhesion molecules
2.74	0.0063	Activating transcription factor 3 long isoform	Proapoptotic, antiproliferative, and anti-invasive effect
2.58	0.011	Small inducible cytokine subfamily A	Antiproliferative or proliferative effect (cell type specific)

Next, we investigated the effect of inhibition of S100A9 expression on the invasive potential of OS cells. Figure 4(A) shows a representative image of MG63 cells invading through the Matrigel (40 µg)-coated membrane at 48 h after transfection with control siRNA or siRNA1/2 against S100A9. The number of invading cells was significantly reduced in cells transfected with siRNAs against S100A9(vs control siRNA, P = 0.026 and P = 0.017). Transfection with siRNA2 decreased the number of invading cells to 39.6% of that in control siRNA transfected cells (Figure 4(B)). The same analyses (Matrigel, 20 µg; incubation time, 72 h) were performed in U2OS cells with similar results (Figure 4(C), P = 0.018).

The results of the proliferation assay showed no significant difference in proliferation at 2 days after transfection with siRNA against S100A9 in MG63 cells and at 3 days in U2OS cells (Figure 3(C)). Therefore, it is unlikely that the decreased invasiveness caused by transfection of siRNA against S100A9 reflects a decrease in the number of cells in siRNA-treated cultures. The invasive behavior of cancer cells in the *in vitro* invasion assay was thought to be independent of proliferative activity.

### Global change in gene expression patterns induced by inhibition of S100A9

To elucidate the molecular mechanism underlying the function of S100A9, we examined global changes in gene expression in MG63 cells at 48 h after transfection with siRNA against S100A9 or control siRNA. To identify specific genes that may be regulated by S100A9, we screened for transcripts with at least twofold differences in signal intensity (false discovery rate for twofold difference, 1.41%). We eliminated transcripts whose detection calls were absent to minimize detection of false upregulation or downregulation. Of the 17,086 transcripts analyzed, 98 (0.57%) showed a twofold downregulation in S100A9 siRNA-transfected cells compared with control siRNA-transfected cells, and 119 (0.69%) transcripts showed a twofold upregulation in S100A9 siRNA transfected cells. Most of the genes identified as differentially expressed were not reported previously in association with S100A9 expression. Nine known genes upregulated or downregulated by S100A9 knockdown are listed in Tables 1 and 2. Several genes downregulated or upregulated by S100A9 knockdown were associated with proliferative properties or apoptosis or invasion of cancer cells. Therefore, the upregulation of these genes may lead to the inhibition of cancer cell motility or invasion. These findings suggest that proliferation or invasion/metastasis-promoting genes and growth or tumor suppressor genes may be direct or indirect targets of S100A9 activity.

**Table 2.** List of nine downregulated genes after transfection of siRNA against S100A9.

Fold change	*p*	Genename (symbol)	Putative cellular function
0.324	0.0058	Mutant cyclin-dependent kinase-associated protein phosphatase	Cell cycle regulator
0.387	0.0037	Matrix metallopeptidase 1 (MMP1)	Invasive effect
0.534	0.018	Solute carrier family-16 (SLC16A2)	Apoptotic effect
0.362	0.004	Laminin gamma 2 (LAMC2)	Invasive effect
0.084	0.0081	S100 calcium-binding protein A12 (S100A12)	Invasion and migration
0.387	0.013	Laminin gamma 2 (LAMC2)	Invasion and migration
0.452	0.038	Sulfatase 1 (SULF1)	Invasion and migration
0.414	0.016	Toll like receptor 4 (TLR4)	Proliferation, invasion and apoptosis
0.376	0.0084	Centromere protein A	Related tomitotic behavior of chromosomes

## Discussion

This is the first report describing the quantitative analyses of S100A9 mRNA levels in OS tissues and microdissected OS cells from patients with OS. S100A9 mRNA expression levels were significantly higher in tumor than in normal bone tissues. Microdissection-based quantitative analysis of mRNA levels revealed that S100A9 was differentially expressed in OS tissues and normal bone tissues. These results were confirmed by immunohistochemistry, suggesting that stepwise upregulation of S100A9 is related to carcinogenesis in OS, and that accurate quantification of S100A9 could be used as a screening test in individuals with radiologic evidence of OS. This suggests that quantitative analysis of mRNA is a promising diagnostic tool for identifying OS in clinical specimens, provided that target mRNA expression levels differ sufficiently between malignant and nonmalignant cells.

Previous studies reported differential expression of S100A9 in non-small cell lung cancer [11], breast cancer [12,13], gastric cancer [14], and colorectal cancer [15] by immunohistochemistry, consistent with the results of the present immunohistochemistry and microdissection-based mRNA analyses of OS and normal bone tissues. Therefore, differential expression of S100A9 in OS and normal tissues suggests that quantitative analysis of S100A9 mRNA levels in OS may be a promising modality for detecting OS.

Although S100A9 expression has been analyzed previously, the role of S100A9 is not well understood. In the present study, RNAi experiments clearly showed that S100A9 is involved not only in cell proliferation but also in the invasion of OS cells. Oligonucleotide microarray data revealed that inhibition of S100A9 affects the expression of certain genes, such as toll-like receptor 4 (TLR4) and activating transcription factor 3 long isoform, which are involved in cell proliferation and invasion. Cheng et al. [9] and Khammanivong [16] showed that S100A9 regulates cell cycle progression or proliferation, consistent with the present proliferation assay data. We showed that inhibition of S100A9 expression suppressed the invasive potential as well as proliferation in OS cell lines. Cross et al. [17] and Hibino et al. [7] reported differential expression of S100A9, as determined by immunohistochemistry or tissue microarrays, in cancers with or without metastasis. These data suggest that S100A9 is of clinical value for the diagnosis and staging of OS and for determining prognosis. Interference with the multiple activities of S100A9 is an attractive strategy for the treatment of OS.

In conclusion, the results of expression analyses suggest that S100A9 is upregulated in OS, and that quantification of S100A9 mRNA may be a useful tool for the diagnosis of OS. The present data also indicate that S100A9 may be a promising diagnostic marker and therapeutic target in OS.

## Research highlights

1. S100A9 is overexpressed in osteosarcoma.
2. S100A9 is a useful diagnostic marker for osteosarcoma.
3. S100A9 is a promising therapeutic target for osteosarcoma.
